# Comparative analysis of selected methods of carbapenemase determination among clinical *Klebsiella pneumoniae*

**DOI:** 10.1371/journal.pone.0318852

**Published:** 2025-02-07

**Authors:** Agata Pruss, Alicja Skierska, Paweł Kwiatkowski, Helena Masiuk, Joanna Jursa-Kulesza, Stefania Giedrys-Kalemba, Iwona Bilska, Monika Sienkiewicz, Maria V. Melnyk, Barbara Dołęgowska

**Affiliations:** 1 Department of Laboratory Medicine, Chair of Microbiology, Immunology and Laboratory Medicine, Pomeranian Medical University in Szczecin, Szczecin, Poland; 2 Department of Diagnostic Immunology, Chair of Microbiology, Immunology and Laboratory Medicine, Pomeranian Medical University in Szczecin, Szczecin, Poland; 3 Department of Clinical Microbiology, Chair of Microbiology, Immunology and Laboratory Medicine; Pomeranian Medical University in Szczecin, Szczecin, Poland; 4 Microbiological Laboratory, Independent Public Clinical Hospital No. 1 in Szczecin, Szczecin, Poland; 5 Department of Pharmaceutical Microbiology and Microbiological Diagnostic, Medical University of Łódź, Łódź, Poland; 6 Department of Epizootology, Microbiology and Virology, National University of Life and Environmental Sciences of Ukraine, Kyiv, Ukraine; University of Nevada, Reno School of Medicine, Nevada State Public Health Laboratory, UNITED STATES OF AMERICA

## Abstract

*Klebsiella pneumoniae* is a typical opportunistic pathogen that exhibits multiple virulence factors and antibiotic resistance conditioning mechanisms. Carbapenemases are enzymes that help bacteria to exhibit the strongest resistance against antibiotics. Therefore, in routine microbiological diagnoses, it is crucial to confirm antibiotic-resistant strains, including carbapenemase-producing bacteria strains, isolated from patients. Two types of tests play an important role here: phenotypicand molecular diagnostic methods. The latter complement phenotypic tests and a mandatory procedure to confirm the detection of carbapenemases. This study aimed to evaluate the usefulness and effectiveness of tests and methods used to identify and confirm the ability of clinical *K*. *pneumoniae* strains to produce carbapenemases. The production of carbapenemases was assessed using phenotypic and genetic methods. The strains tested showed complete resistance to most beta-lactams and varying sensitivity to drugs from the quinolone carbapenem group and aminoglycosides. Among the most commonly produced carbapenemases were the metallo-beta-lactamase (NDM) family. The most accurate phenotypic method for detecting carbapenemases was the NG CARBA-5 assay, and the PCR method confirmed these results. Notably, a few inconclusive results were obtained for NDM-positive and VIM-positive strains when the disk diffusion method and CIM test were used. Further, the Carba tube assay and the RAPIDEC CARBA NP assay produced questionable results for the OXA-48 strain group. This group also generated false-negative results on Carba’s CHROM ID medium.

## Introduction

*Klebsiella pneumoniae* is a pathogen well-known for its prevalence and virulence in infection medicine. In humans, it is considered as an opportunistic pathogen. From the sites it colonizes, it can enter the bloodstream or adjacent tissues, causing infection [1]. It is considered lethal due to its virulence and antibiotic resistance.

*K*. *pneumoniae* is an etiological agent of life-threatening infections such as lower respiratory tract infections, wound infections, bacteremia, septicemia, and urinary tract infections [1–3]. Their resistance mechanisms render them insensitive to many antibiotics. Drugs of last resort, namely antibiotics from the carbapenem group, are increasingly failing to have the intended effect. The production of carbapenemases determines resistance to carbapenems andother beta-lactam antibiotics [4]. Carbapenemase-producing strains most often show resistance to other drug groups as well.

Carbapenemases are the most versatile family of beta-lactamases. They hydrolyze almost all antibiotics belonging to the beta-lactam group, as well as do not yield to the inhibitory effect of beta-lactamase inhibitors [5]. They are mainly plasmid-encoded, associated with a high risk of transmission between species, mainly among *Enterobacteriaceae* [6]. *K*. *pneumoniae* is the primary producer of New Delhi metallo-beta-lactamase (NDM), but it can also produce other enzymes of this group, such as the VIM-type (Verona Integron encoded metallo-beta-lactamase) and IMP-type (imipenemase, active against imipenem). The rapid spread of *K*. *pneumoniae* NDM has been observed in India and other Asian countries, hospitals, and the environment. The *bla*_NDM_ genes are located in plasmids with high conjugation potential, which, with the help of a horizontal transfer, causes rapid spread among the bacteria [6,7]. KPC (*Klebsiella pneumoniae* carbapenemases) are class A carbapenemases first detected in *K*. *pneumoniae* but have also spread to other members of the *Enterobacteriaceae*. Another type of carbapenemase produced by *K*. *pneumoniae* belongs to class D, the OXA-48 enzymes [7].

The identification of carbapenemases is of immeasurable importance in routine microbiological diagnosis. It is crucial for developing and executing the treatment plan and activating the infection control mechanism. Meticulous diagnostics are extremely useful in epidemiological studies that contribute to limiting the spread of isolates producing these enzymes. Two types of tests play an essential role here—phenotypic and molecular diagnostic [8].

Phenotypic diagnosis is based on the classification of bacteria based on their characteristics. The ability to produce carbapenems is assessed, among other things, by methods that detect the change in pH after hydrolysis of the beta-lactam ring or the hydrolysis products of carbapenems [6]. Selective media and discs containing antibiotics are also used. The results obtained by phenotypic methods should be verified using molecular biology tests [8]. The first step in the algorithm for diagnosing carbapenemase-producing isolates is screening tests. In the case of reduced sensitivity of the tested strain to any of the carbapenems, particularly meropenem and ertapenem, according to the European Committee on Antimicrobial Susceptibility Testing (EUCAST) recommendations, such microorganisms should undergo diagnostic identification of carbapenemases [9]. Screening tests are based on the disk-diffusion method. These methods compare the activity of drugs with and without inhibitors. With these methods, it is possible to obtain information about the type of carbapenemase that a particular strain produces [10]. EUCAST does not recommend MHT (Modified Hodge Test) because of its low specificity and complex interpretation of results [11]. On the other hand, MHT has good sensitivity to many other carbapenemases, such as OXA-48, or enzymes similar to VIM and IMP [10]. CARBA NP is a test developed in 2012 by a team of French researchers [12]. It has been adopted by the Clinical and Laboratory Standards Institute (CLSI) as one of the standard phenotypic methods for detecting carbapenemases. Other assays have been developed based on the same regimen, including the RAPIDEC CARBA NP assay or the Blue-Carba assay [13]. In 2015, a team of Dutch researchers described a new method for identifying carbapenemases among bacilli, including *K*. *pneumoniae*. The CIM test (carbapenem inactivation method) is characterized by its low price, ease of performing and reading the results, and high sensitivity and specificity. Researchers emphasize, however, that results obtained with the CIM test should always be interpreted by comparing the results of other methods [14]. The advent of immunochromatographic tests has accelerated the diagnostic detection of carbapenemases. One example is the NG-TEST CARBA. This method was developed for rapid detection of five major carbapenemases, including KPC, OXA-48, NDM, VIM, and IMP [15]. It is based on detecting specific epitopes of these enzymes [16]. Chromogenic media can support the identification of carbapenemase-producing strains. Specific colony staining helps identify the resistance mechanism of strains. The use of selective media is characterized by ease of use, as these are single-step procedures. However, when interpreting the results, they should be compared with those obtained by other methods [17]. Molecular biology methods (such as Xpert Carba-R) have greater sensitivity and precision compared to conventional methods. They perfectly complement phenotypic tests and are a mandatory procedure to confirm the detection of carbapenemases [18].

This study aimed to evaluate the usefulness and effectiveness of tests and methods used to identify and confirm the ability of clinical *K*. *pneumoniae* strains to produce carbapenemases.

## Materials and methods

### Strains and culture conditions

A total of 138 *K*. *pneumoniae* strains belonging to the Department of Microbiology, Immunology, and Laboratory Medicine of the Pomeranian Medical University in Szczecin collection, isolated between June 2022 and June 2023 from clinical materials of patients of three different hospitals, were used in this study. Strains were isolated from blood, bronchoalveolar lavage, urine and wound swab. All samples were cultured on Columbia agar supplemented with 5% sheep blood (bioMérieux, Warsaw, Poland) and incubated overnight at 37°C in an aerobic atmosphere. The strains were identified using the MALDI-TOF MS method (Brücker, Germany). This research does not require an ethics statement.

### Clonal diversity of *K*. *pneumoniae* strains

To demonstrate the clonal diversity of the analyzed *K*. *pneumoniae* strains, they were subjected to molecular typing by pulsed-field gel electrophoresis (PFGE) using CHEF Bacterial Genomic DNA Plug Kits (Bio-Rad, Marnes-la-Coquette, France) [19].

### Drug susceptibility

The sensitivity of the strains to selected antibiotics and chemotherapeutics was determined using the disk-diffusion method [Becton Dickinson, USA]. The values obtained were assigned to aselected sensitivity category based on the EUCAST recommendations [9].

### Phenotypic screening determination of carbapenemases

Control strains were used for all tests. *K*. *pneumoniae* ATCC 25955 as negative control, *K*. *pneumoniae* ATCC 700603 as MBL positive control, *K*. *pneumoniae* NCTC 13438 as KPC positive control, *K*. *pneumoniae* NCTC 13442 as OXA-48 positive control, *K*. *pneumoniae* BAA 2146 as NDM positive control and *Enterobacter cloacae* JMI10526 as IMP positive control. The phenotypic determination of carbapenemases was performed using the disk-diffusion method [10]. DDST-EDTA (double-disk synergy test) for MBL, CDT (combined disc test) for KPC, and TEM for OXA-48 were performed.

### Confirmation of carbapenemase production

#### Carbapenem Inactivation Method (CIM)

The test was performed according to the literature [14]. In the case where the test isolate did not produce carbapenemase, a prominent observation was a clear large zone of growth inhibition around the meropenem disc; in the case of the carbapenemase-producing strain, a marked reduction in the zone of growth inhibition was observed.

#### CARBA tube test (Diagnostics, Slovakia)

The CARBA tube test confirmed that the tested strain produced enzymes from the carbapenemases group. The test was performed per the manufacturer’s instructions [20].

#### RAPIDEC CARBA NP test (bioMerieux, France)

The RAPIDEC CARBA Carba NP assay was a simplified version of the Carba test that allowed confirmation of carbapenemase production by the test strain [13].

#### NG CARBA-5 test (BioTech)

This rapid immunochromatographic method was used to confirm the presence or absence of KPC, NDM, VIM, OXA-48, and IMP-type carbapenemases [15].

#### Carba’s CHROM ID (bioMérieux, France)

The strains were seeded on a chromogenic medium. The growth of blue colonies indicated that the tested bacterial strains produced carbapenemases [19].

#### Xpert Carba-R (Cepheid, Sweden)

The test was performed by Real-Time PCR using the Xpert Carba-R cassette. The test confirmed the presence of genes encoding carbapenemases KPC, NDM, VIM, OXA-48, and IMP [18].

## Results

### Drug susceptibility of the tested strains

This study reported complete resistance to antibiotics from the penicillin group, penicillins with beta-lactamase inhibitors, and cephalosporins. The sensitivity of isolates to drugs from the carbapenem group, quinolones, and sulfonamides varied. The highest percentage of sensitivity was obtained for drugs from the aminoglycoside group (Table 1).

**Table 1:** Drug susceptibility of *K*. *pneumoniae*.

Antibiotic	Antibiotic Concentration [μg]	Resistant *n* (%)	Susceptible *n* (%)
Amoxicillin with clavulanic acid	20/10	138 (100)	0 (0)
Piperacillin/tazobactam	30/6	138 (100)	0 (0)
Piperacillin	30	138 (100)	0 (0)
Ceftazidime	10	138 (100)	0 (0)
Cefotaxime	30	138 (100)	0 (0)
Cefepime	30	138 (100)	0 (0)
Gentamicin	10	114 (82.6)	24 (17.4)
Amikacin	30	102 (73.9)	36 (26.1)
Ciprofloxacin	5	124 (89.8)	14 (10.1)
Imipenem	10	121 (87.6)	17 (12.3)
Meropenem	10	138 (100)	0 (0)
Ertapenem	10	138 (100)	0 (0)
Trimethoprim/sulfamethoxazole	1.25–23.75	129 (93.5)	9 (6.5)

### Detection of carbapenemases by disk-diffusion method

**Fig 1:**
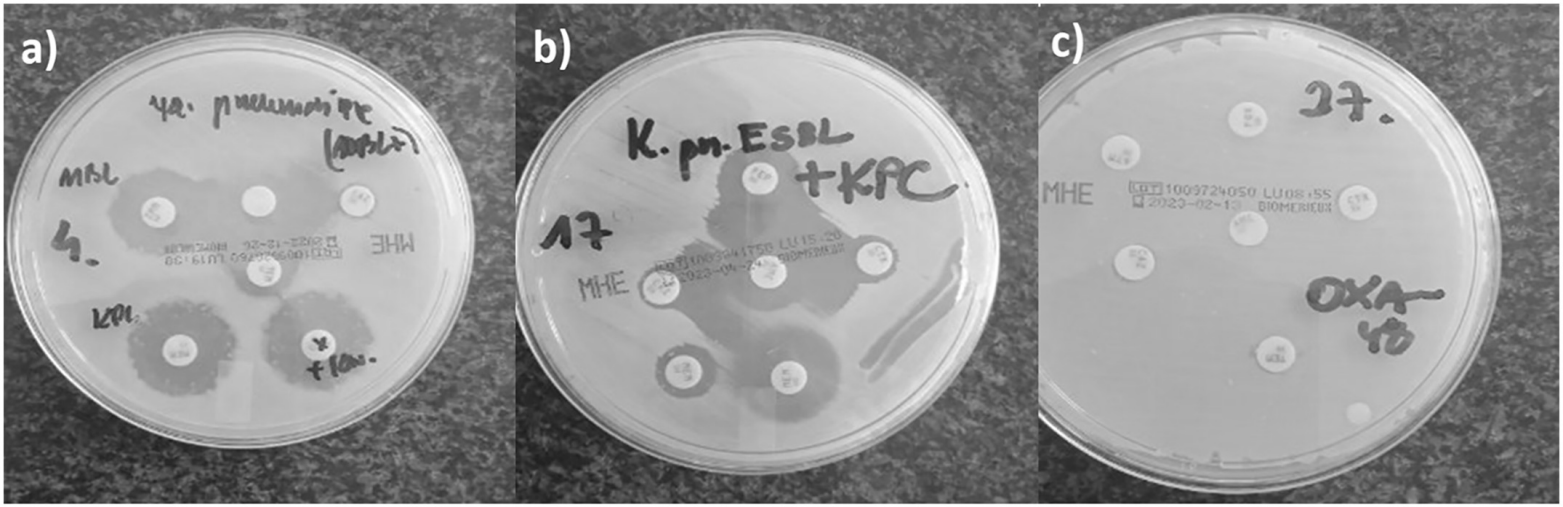
An example of a positive results in the disk-diffusion method.

The DDST-EDTA method was used to confirm the production of MBL-type carbapenemase in 60 out of 138 strains (Fig 1). For six strains, the result was difficult to interpret. In the CDT test, 36 out of 138 strains tested positive, indicating KPC production. Using a temocillin disc, 13 of 138 strains suspected of producing OXA-48 were selected.

### CIM test results

**Fig 2:**
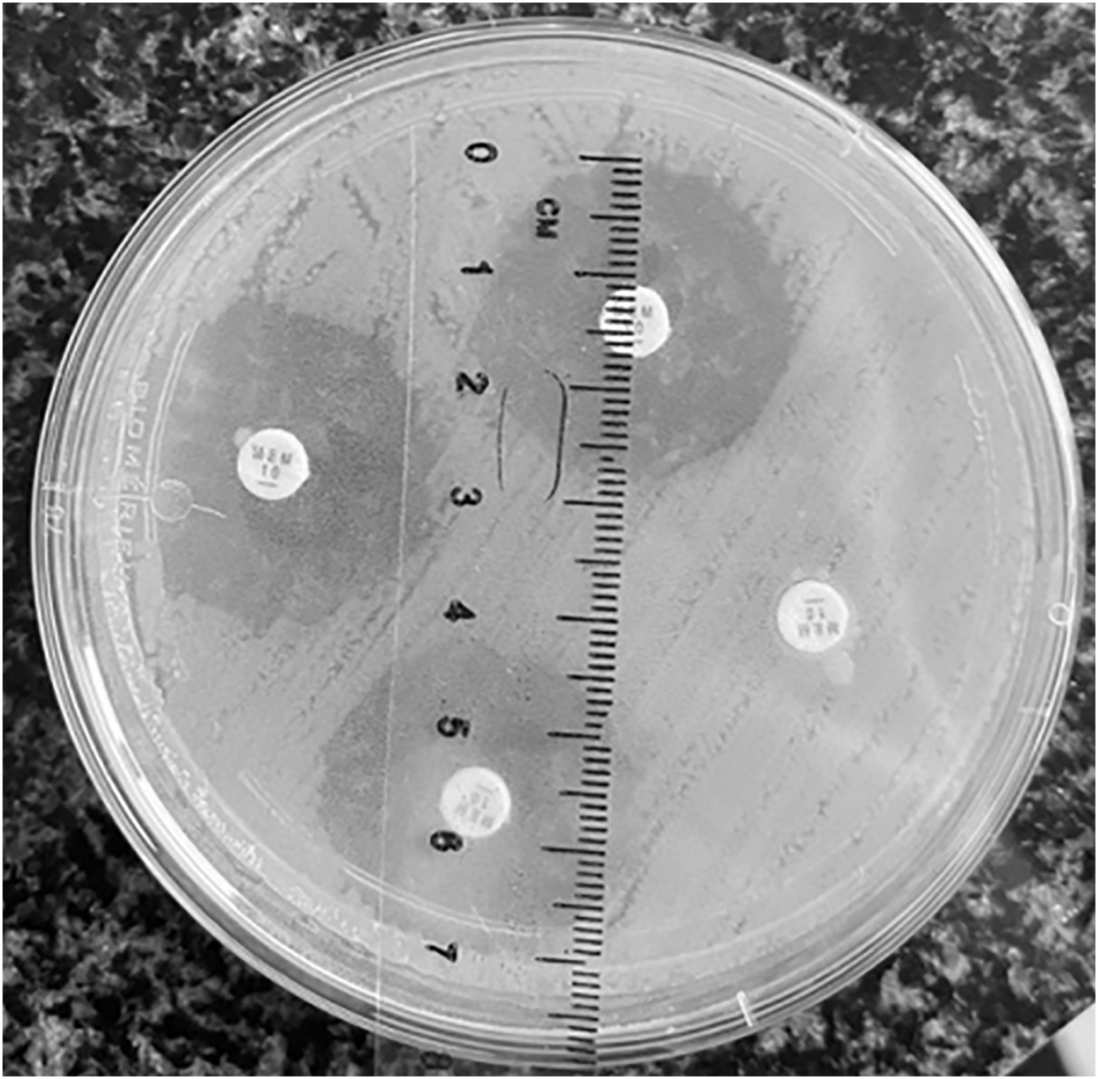
An example of a positive and negative result in the CIM test.

In the CIM test, the vast majority (112; 81.1%) of the tested *K*. *pneumoniae* showed a complete absence of growth zone inhibition, indicating a positive result (Fig 2). An uninterpretable result was obtained for three strains; a negative result was observed in 23 strains.

### CARBA tube test results

**Fig 3:**
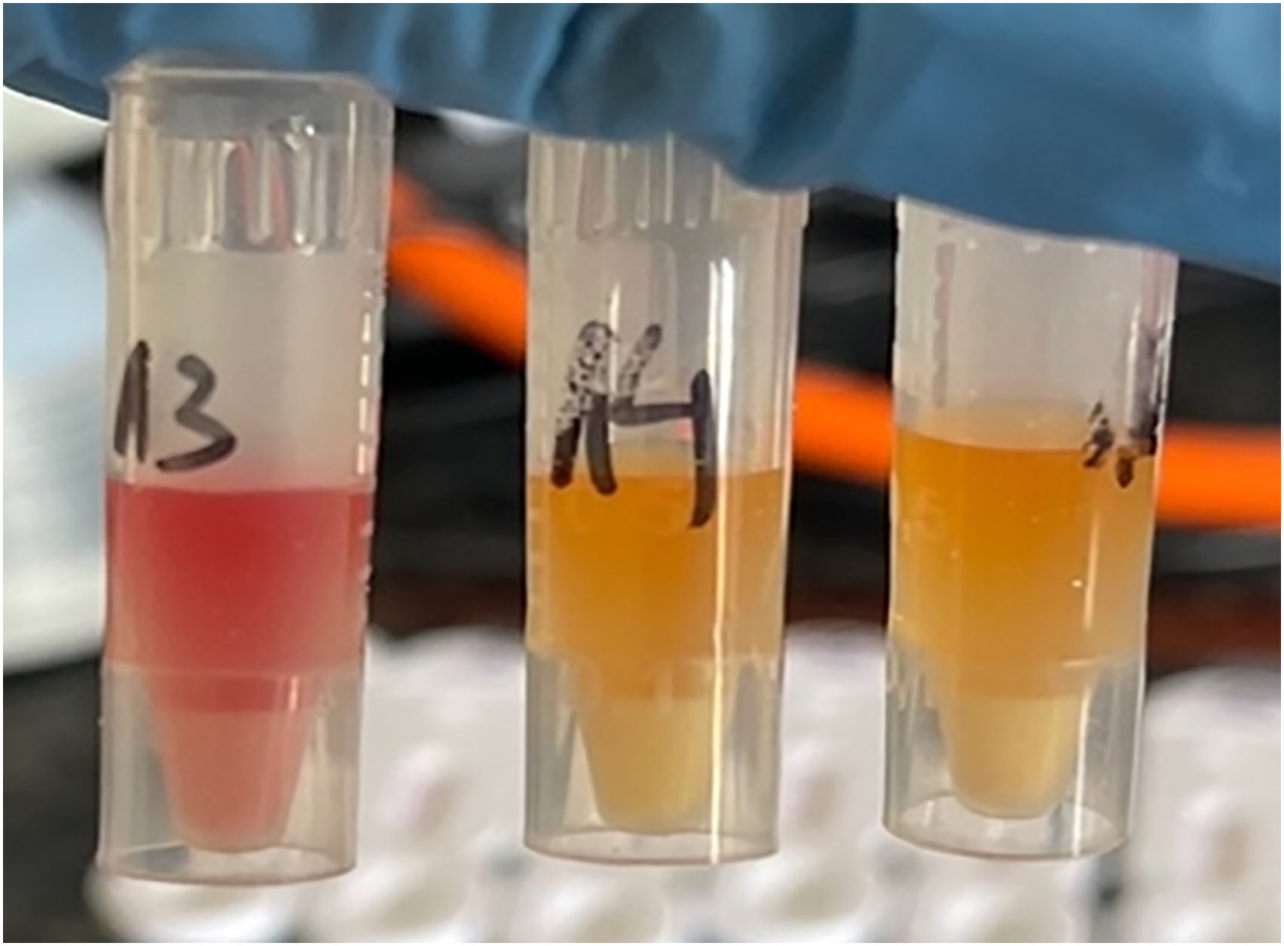
Comparison of a negative CARBA test result (left tube) with a positive CAR-BA test result (middle and right tubes).

The CARBA test obtained a positive result in 107 (77.5%) cases, identifying the tested bacterial strain as producing carbapenemases (Fig 3). A total of 23 (16.6%) isolates (7.9%) were identified as those that do not produce carbapenemases. In 8 cases (5.8%), the result was questionable and difficult to interpret.

### RAPIDEC CARBA NP test results

**Fig 4:**
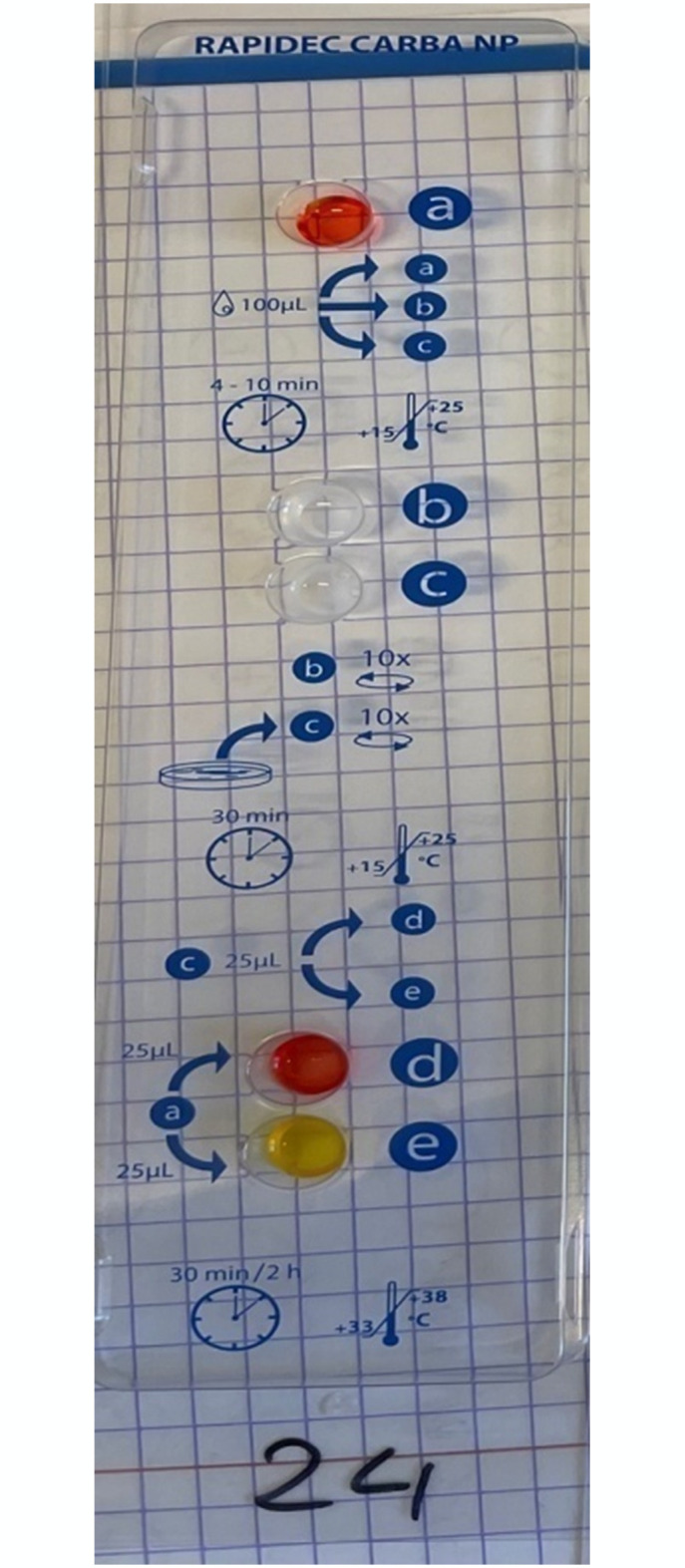
An example of a positive result of RAPIDEC CARBA NP test.

The RAPIDEC CARBA NP test yielded results identical to that of the CARBA test; however, in two cases, the results were questionable results for other strains (Fig 4).

### Carba’s CHROM ID

**Fig 5:**
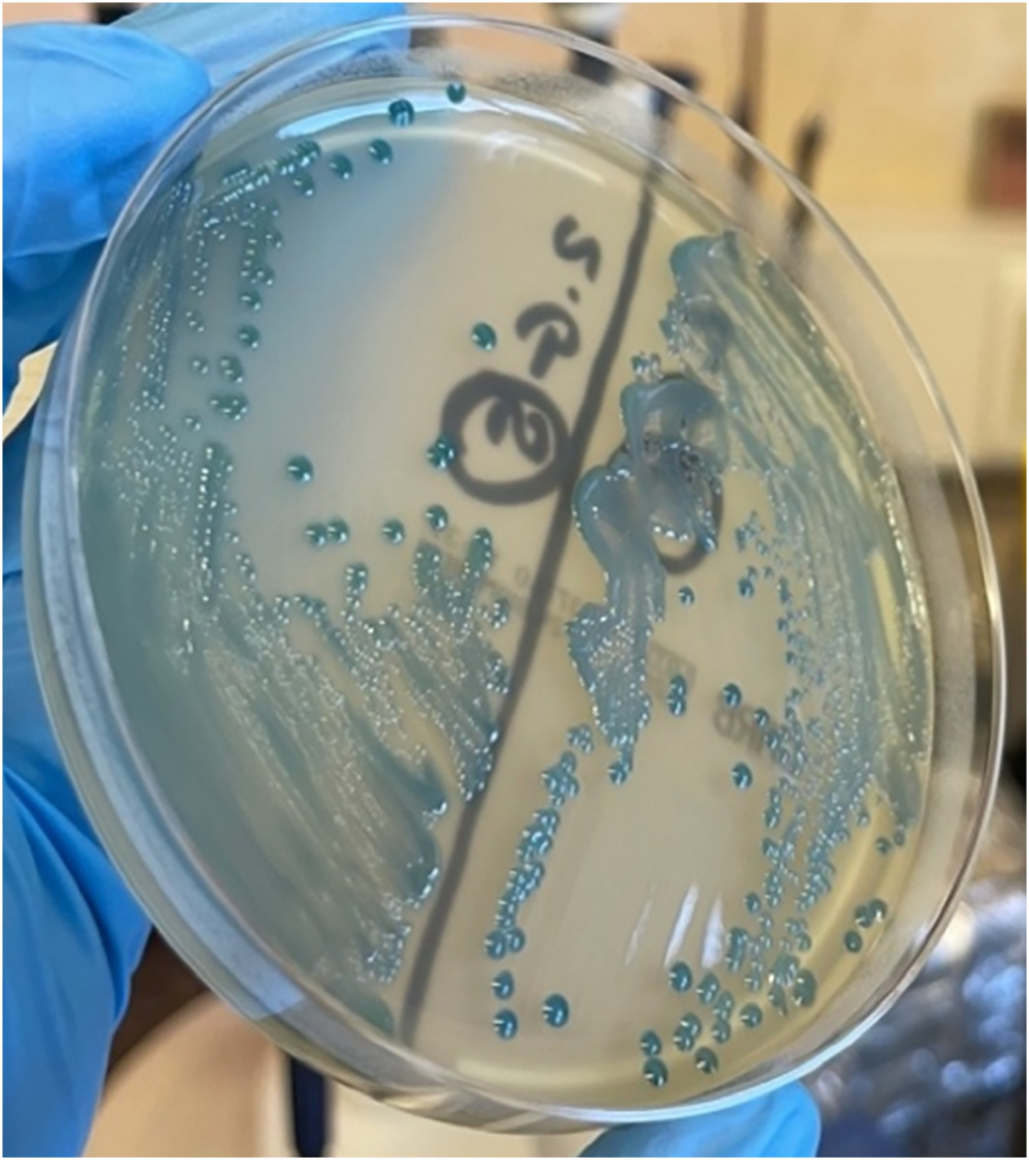
An example of a positive result of Carba’s CHROM ID.

A total of 107(77.5%) *K*. *pneumoniae* strains grew blue colonies on the Carba’s ID medium, which were identified as those that produce carbapenemases (Fig 5). Further, 31 (22.5%) strains were not found to grow on the medium.

### NG CARBA-5 test results

**Fig 6:**
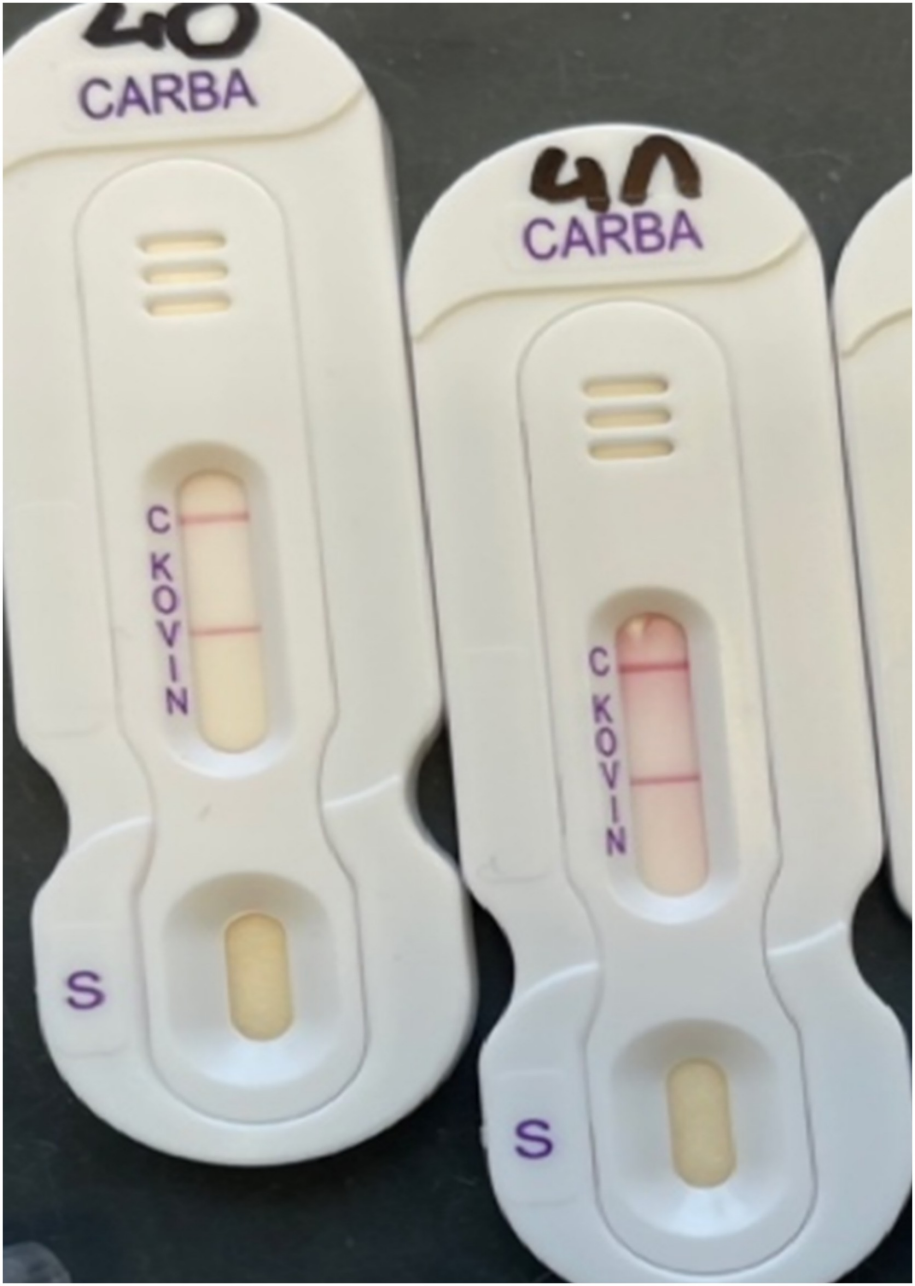
An example of a positive NG CARBA-5 test result.

The NG CARBA-5 test confirmed that among the 138 *K*. *pneumoniae* tested, the vast majority, 54 (39.1%), were strains that produce NDM-type carbapenemase (Fig 6). KPC-type carbapenemase in 36 (26.1%), OXA-48 in 13 (9.4%), and VIM in 12 (8.7%) strains were identified. Notably, 23 (16.6%) of the strains tested were negative. It is worth emphasizing that IMP carbapenemase was not detected in any strain.

### Xpert Carba-R results

**Fig 7:**
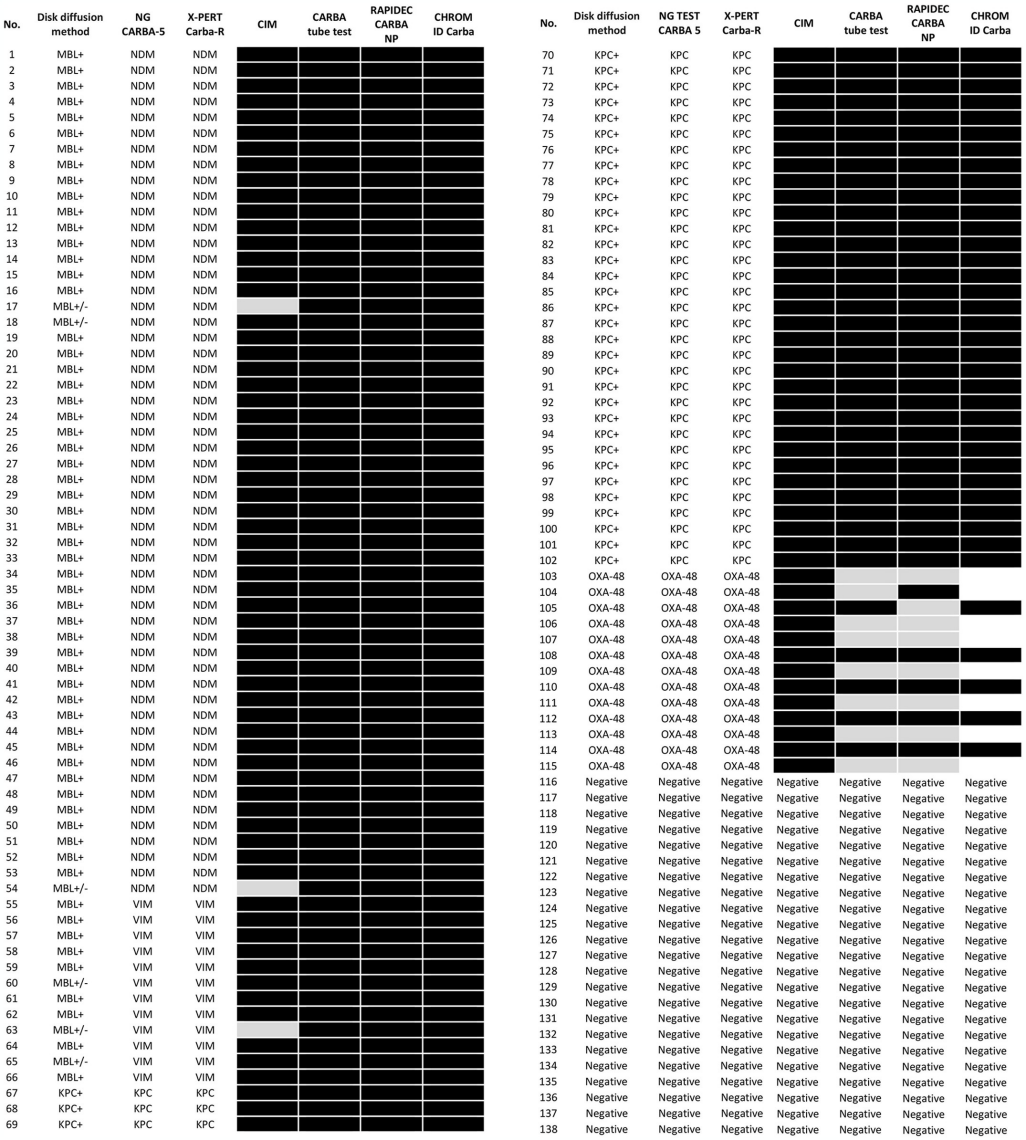
Summary of the results of the carbapenemase assay. Black color—positive result; gray color—doubtful result; white color—negative result.

Using Xpert Carba-R Real-Time PCR, genes encoding individual carbapenemases were detected. The results obtained by this method were identical to that of the NG CARBA-5 test. A summary of the results of the carbapenemase production is shown in Fig 7.

## Discussion

The ubiquity and spread of *K*. *pneumoniae* is a global problem. The vast majority of *K*. *pneumoniae* strains, in addition to numerous virulence factors, have developed resistance mechanisms, which render them resistant to many antibiotics. In an increasing number of isolated strains, various resistance mechanisms have been confirmed, attributed to the increasing production of carbapenemases. This significantly narrows the therapeutic options. Therefore, it is vital to detect them at the earliest possible stage of diagnosis. As carbapenemases exist in several different forms of enzymes, identifying them is not simple. Nevertheless, many tests have been designed to confirm the presence of carbapenemases. At the beginning of diagnostics, tests determine only whether the tested bacterial strain produces enzymes of the carbapenemases group. However, in later stages, more specialized methods are used to determine the types of these enzymes. In Poland, reference methods following the EUCAST guidelines are often used to detect carbapenemases in all strains showing reduced sensitivity to carbapenems. To accelerate and improve the diagnostic detection of carbapenemase-producing pathogens, new tests are being developed every year, the effectiveness and accuracy of which are evaluated in this paper.

There are several phenotypic methods for determining whether a test strain produces carbapenemases. They find their use in the initial stages of diagnostics when it is necessary to identify multidrug-resistant bacteria. Application of these methods facilitates the identification of multidrug resistant bacteria at the initial stages of microbiological diagnostic. Methods described are widely used in routine diagnostic and are relatively simple to perform improving the analysis considerably.

Using the disk-diffusion method, it is possible to test for MBL, KPC, and OXA-48 carbapenemases [21]. While we obtained unequivocal results on the CDT and temocillin tests, the DDST-EDTA test for 6 of the 66 strains yielded a questionable result. In such cases, other tests are recommended to confirm the production of metallo-beta-lactamases [22].

In Poland, the CIM test is particularly recommended because of the high sensitivity and specificity of the results obtained and its low cost using essential laboratory equipment. Many researchers use it to diagnose infections with pathogens suspected of producing carbapenemases. Aguirre-Quinonero *et al*., in their analysis using the CIM test, identified all strains producing carbapenemases of the KPC, NDM, VIM, IMP, and OXA-48 types, obtaining a zone of growth inhibition of about 6 millimeters. They obtained false-negative results for 11 (50%) Guiana-Extended-Spectrum(GES)-6-positive pathogens, and two (17%) isolates not producing carbapenemases in the CIM test came out as false positives. The researchers rated the sensitivity and specificity of the test as sufficient, at 85.7% and 95.7%, respectively [23]. In contrast, a low detection rate of 16.7% was reported for OXA-48 isolates. Of all GES carbapenemase-producing strains, a positive CIM test result was obtained in 75% of cases [24]. In contrast, Crowe *et al*. identified as many as 64 (95.5%) of 67 carbapenemase producers using the CIM test. The researchers detected all strains producing carbapenemases of the KPC, NDM, IMP, and OXA-48 types, with less satisfactory results for VIM isolates (33.3%). The CIM test proved reliable for strains producing several types of carbapenemases, detecting all such pathogens [25]. In this study, carbapenemases were also detected in most strains, but, a questionable result was obtained for three pathogens. Researchers consider the CIM test to be an effective, inexpensive, and simple tool for detecting the most common carbapenemases. However, they highlight unsatisfactory results for OXA-48 carbapenemases, to determine which other methods are recommended. One of them is the CARBA test. It is a rapid test that identifies carbapenemase-producing pathogens. In a study by Wang *et al*., of all the carbapenemase-producing strains, up to 99.31% of isolates were correctly identified using this test [26]. Tijet *et al*. correctly detected carbapenemase-type KPC and VIM in another analysis. NDM carbapenemase-producing strains were positive for 94%, while IMP-type enzymes were positive for 86%. The OXA-48-type carbapenemase-producing strains proved the most difficult to identify, with only 23 (59%) of 39 isolates detected [27]. Similar results were obtained by Morey *et al*. with positive results for KPC, VIM, NDM, IMP, and *Serattia marcescens* enzyme (SME)-type carbapenemase-producing strains. In contrast, they obtained a negative result for all OXA-48-positive pathogens [28]. This study obtained a doubtful result on the CARBA test for eight strains in which carbapenemase and other methods confirmed OXA-48. A positive result was obtained for all other carbapenemase-positive strains included in the study.

Another phenotypic method that identifies carbapenemase-producing strains is the RAPIDEC CARBA NP test. It is often used in microbiology laboratories due to its ease of use. Dortet *et al*. compared several phenotypic methods, including RAPIDEC CARBA NP. Using this test, they detected as many as 94 (99%) out of 95 total carbapenemase-producing pathogens. However, they failed to identify one OXA-48-like carbapenemase-producing strain [29]. In contrast, Mancini *et al*. detected as many as 104 (93.7%) out of 111 total carbapenemase-producing strains using this assay. All KPC-positive, NDM-positive, VIM-positive, IMP-positive, and OXA-48-positive isolates were correctly identified with a positive test result. Kour *et al*. also found positive test results for all 26 isolates tested [30]. Differing results, however, were obtained for strains producing the OXA-48 carbapenemase type. Only 24 (77.4%) out of 31 total strains were determined to be positive. Moreover, all strains producing the less common FRI-1, SME, and IMI-type carbapenemases were detected using this method [31]. Similar results were obtained by Garg *et al*., who identified as many as 46 (92%) out of 50 carbapenemase-producing strains. They documented that using the RAPIDEC CARBA NP assay, carbapenemase activity was not detected in three OXA-48 positive strains and one IMP positive strain. They also obtained two false positives for carbapenem-resistant strains. In contrast, they obtained negative test results for all carbapenem-sensitive strains [32]. Interestingly, in the current study, a doubtful result was obtained for eight pathogens tested, which correlated with the results of the CARBA test, as this applied only to strains in which other methods detected OXA-48. Furthermore, as in the CARBA test, a positive result was obtained for the other producers of carbapenemases. Given the above, the need for further research using the RAPIDEC CARBA NP test seems obvious. In addition, this is supported by some advantages of this method, which undoubtedly include low cost and ease of use. However, the researchers unanimously noted the unsatisfactory results of this test in detecting strains producing carbapenemases of the OXA-48 family.

Chromogenic substrates are often used in the identification of carbapenemases. One of them is the Carba’s CHROM ID. Wilkinson *et al*. compared four substrates for detecting carbapenemase-producing strains and obtained one of the highest sensitivity (91%) using the Carba’s CHROM ID. Among carbapenemase-producing strains such as IMP, KPC, and VIM, 100% were positive. For the NDM carbapenemase, 85 (96.5%) strains out of 88 grew blue colonies, and for OXA-48 strains, positivity was achieved in only 86.7% of cases [33]. Similarly, Vrioni *et al*. found one of the highest sensitivity and specificity, 92.4% and 96.9%, respectively, for Carba’s CHROM ID among all the substrates tested. Using this medium, they detected as many as 85 (92.4%) out of 92 strains that produced carbapenemases. Notably, the most difficult was the identification of OXA-48 carbapenemase-producing strains, which they only detected using Carba’s CHROM ID with a higher column of bacterial medium [34]. In contrast, Lund *et al*., using a medium, failed to identify 4 (16%) of 25 carbapenemase-producing strains. As with Wilkinson *et al*. and Vrioni *et al*.’s study, OXA-48-positive pathogens were the most difficult to detect. As many as 2 (66.7%) of 3 strains of this genus failed to grow on the Carba’s CHROM ID [35]. In this study, satisfactory results were obtained. All carbapenemase-producing pathogens of the NDM, KPC, and VIM types grew blue colonies on the Carba’s CHROM ID. In the OXA-48 strains, no growth was observed for 8 of the 13 isolates. No growth was observed for isolates that did not produce carbapenemases. All the authors mentioned above concluded that the Carba’s CHROM ID is a valuable tool, producing satisfactory results in detecting carbapenemase-producing strains. Noteworthily, OXA-48-positive strains posed the most significant difficulty in identification in all studies.

One of the most widely used tests is the NG CARBA-5. It is a specialized method that allows one to identify within 15 minutes the type of carbapenemase produced by the tested strain. Han *et al*. confirmed the production of carbapenemases of the KPC, NDM, OXA-48, IMP, and VIM types. With more than 100% specificity and sensitivity, they detected bacterial strains producing two carbapenemases simultaneously, obtaining two red lines in the test area. These isolates produced KPC-2 and NDM-1, KPC-2 and NDM-5, and NDM-5 and OXA-48, while they obtained a negative test result for non-carbapenemase-producing strains [36]. On the other hand, alarming results were obtained by Baer *et al*., as they correctly identified 89.5% of the strains tested, while 10.5% of the isolates showed a false negative result. With a sensitivity of 89.6% and a specificity of 100%, they identified 100% of KPC producers, 80% of NDM, 87.5% of OXA-48, and 83.3% of VIM. These authors also observed that the test failed to detect a single IMP-producing pathogen [37]. High sensitivity and specificity of 100% and 99.9%, respectively, were obtained by Zhu *et al*., who detected all the carbapenemase-producing pathogens of the KPC, NDM, VIM, and OXA-48 types. In the case of one IMP-4 strain, they obtained a false-positive result for NDM, which lowered its specificity to 99.6% [38]. In the current study, satisfactory results were obtained, detecting 100% of strains producing carbapenemases of the NDM, VIM, KPC, and OXA-48 types, and negative results were obtained for strains not producing carbapenemases. These results correlate with the current literature, indicating that the NG CARBA-5 test is an efficient, rapid, reliable, and convenient tool for detecting the most common carbapenemases.

A very effective method for detecting different carbapenemases is the Xpert Carba-R Real-Time PCR method. It relies on confirming the presence of characteristic genes such as *bla*_KPC_, *bla*_IMP_, *bla*_NDM_, *bla*_VIM_, and *bla*_OXA-48_. Cointe *et al*.’s analysis confirmed the excellent detection of these genes, with a sensitivity and specificity of 100%. Using this method, they identified all the strains that produce particular carbapenemases [39]. Moreover, Kost *et al*. confirmed with 100% sensitivity the detection of genes encoding the most common carbapenemases such as KPC, NDM, VIM, IMP, and OXA-48. In contrast, following the researchers’ assumptions, the test did not detect such carbapenemases as GES or SME [40]. The high efficiency of Xpert Carba-R was also documented by Smith *et al*. With 100% sensitivity, they detected and identified all carbapenemase genes in all strains. Further, they had no false positives, indicating 100% specificity [41]. Other authors have also agreed that the Xpert Carba-R test is a reliable and rapid way of detecting five carbapenemases in organisms that produce single and several simultaneous carbapenemases [42]. This research also confirmed the indisputable usefulness of this method. In all strains suspected of producing particular carbapenemases, the presence of the corresponding genes was confirmed. Many researchers have found this method a quick, easy, and susceptible tool for detecting genes that encode the most common carbapenemases.

## Conclusions

In conclusion, identifying carbapenemase-producing strains should be mandatory in view of increasing bacterial resistance to antibiotics. It concerns not only microbiological or clinical issues but also epidemiological ones. Nowadays, several methods and tests are available. Studies have confirmed the effectiveness of phenotypic and molecular methods, and the results obtained through them have been characterized by high sensitivity and specificity. Phenotypic methods are characterized by low-cost simplicity in performance so that they can be used for screening in everyday microbiological diagnosis. However, caution should be exercised in identifying OXA-48 carbapenemase, as it produces the most questionable or false-negative results on phenotypic tests. On the other hand, molecular methods using specialized equipment unquestionably detect genes responsible for the production of carbapenemases and confirm specific resistance mechanisms.

This study proved that only NG CARBA-5 demonstrated broadest capability among the evaluated tests. However, any phenotypic test result should be confirmed by a molecular method (e.g. Xpert Carba-R), detecting specific genes.
